# Decline in HPV-vaccination uptake in Denmark – the association between HPV-related media coverage and HPV-vaccination

**DOI:** 10.1186/s12889-018-6268-x

**Published:** 2018-12-10

**Authors:** Camilla Hiul Suppli, Niels Dalum Hansen, Mette Rasmussen, Palle Valentiner-Branth, Tyra Grove Krause, Kåre Mølbak

**Affiliations:** 1 Department of Infectious Disease Epidemiology and Prevention, Statens Serum Institut, Artillerivej 5, DK-2300 Copenhagen S, Denmark; 2 Department of Epidemiology Research, Statens Serum Institut, Artillerivej 5, 2300 Copenhagen, Denmark; 3 Division of Infectious Disease Preparedness, Statens Serum Institut, 2300 Copenhagen, Artillerivej 5 Denmark; 4 National Institute of Public Health, University of Southern Denmark, Øster Farimagsgade 5A, 1353 Copenhagen, Denmark

**Keywords:** Human papilloma virus vaccine, HPV-vaccine, Immunization, Media monitoring, Vaccination coverage, On-line searches

## Abstract

**Background:**

In 2014, Denmark experienced a rapid decline in vaccination uptake for the human papillomavirus (HPV) vaccine after a successful introduction of the vaccine in 2009. Before the decline, the uptake of the first HPV vaccine was around 90% for girls born in the period 1998 to 2000, while it dropped to 54% for girls born in 2003. The decline followed negative public attention from 2013 coinciding with increasing suspected adverse-event reporting to the Danish Medicines Agency. The aim of this study is to describe the HPV-vaccination uptake, to quantify relevant HPV-related media coverage, and analyse the relation between media coverage and HPV-vaccination acceptance in Denmark in year 2009–2016.

**Methods:**

Three types of data were used for the analysis: Immunisation data from 243,415 girls, media coverage (8524 news items) and Google search activity. We used changes in the correlation between media coverage and vaccination uptake to identify a changing point in their relationship. The relationship before and after the changing point was analysed determined on the interactions between vaccination uptake, media and search activity, with search activity as a proxy for public attention.

**Results:**

We identified July 2013 as a changing point in the relationship between media coverage and vaccination uptake. We found no significant relationship between media coverage and vaccination uptake in the first part of the time series (June 2009 to June 2013), whereas from July 2013 and onwards there was a negative Pearson’s correlation of − 0.52. The changing point coincides with both an increase in Google searches for “HPV side effects” and media coverage with negative content.

**Conclusions:**

Following a successful launch of the HPV-vaccination programme, concerns about vaccine safety shifted the public opinion and the coverage by the media. The noticeable shift in correlation between vaccination uptake and media coverage before and after July 2013 could indicate that increased media coverage influenced the decline in vaccination uptake. Media monitoring may represent an important tool in future monitoring and assessment of confidence in vaccination programmes.

## Background

With nearly 400 women diagnosed with cervical cancer and 100 annual deaths in Denmark (data including 2014), the incidence rate remains high among Western European countries [1]. Accordingly, Denmark was one of the first countries to implement publicly funded vaccination against human papillomavirus (HPV). This was the latest addition to the Danish childhood vaccination programme protecting against nine infectious diseases. The programme is voluntary, offered free of charge and widely accepted. The HPV-programme was launched in 2008 primarily targeting 12-year-old girls with a quadrivalent HPV-vaccine. Effectiveness, impact, and safety of the quadrivalent HPV-vaccine is well established [2].

In the first years after the launch, the HPV-vaccine was received positively resulting in a high uptake (> 90% with at least one HPV vaccination among girls born 1998 to 2000). From 2013, the programme was challenged by an increasing number of reported suspected adverse events [3] and subsequently the media attention increased and shifted in content. The Danish media started to recount case-stories of suspected adverse events from Danish girls and women. This accelerated after a television documentary, March 2015, describing a group of girls with a wide range of disabling symptoms presumed to follow HPV-vaccination. The documentary was widely shared and reported on social and written media. Subsequently an additional number of similar case-stories and anecdotes were disseminated in various media sources, including social media. Until now, no epidemiological study have been able to substantiate an increased risk of the alleged adverse events with a history of HPV-vaccination. Nevertheless, this shift in media attention was followed by a marked decline in HPV-vaccination uptake and an increased rate of reported suspected adverse events [4].

The aim of the present study was to describe the decline in the uptake of HPV-vaccination and to explore the relationship between media coverage, Google searches, and vaccination activity under the hypothesis that media coverage affects search activity and vaccination uptake.

It is known from previous studies that media coverage affects the uptake of vaccinations [5, 6]. Traditionally, it has been very difficult to attain data, which captures the temporal association between external exposure such as media coverage and vaccination uptake [5, 6]. The Danish Vaccination Register [7] contains registration on both personal identifiers, type, and time of vaccination allowing for investigations of timely associations with external factors. We took advantage of this data source to calculate the vaccination coverage per birth cohort and the rate of administered vaccines per month as a measure of vaccination activity. A similar approach has been used in a recent study [8], where the focus was the MMR vaccination uptake and media coverage. We go further in this study by also including data on online search patterns.

Frequencies of web searches has successfully been used for predicting vaccination uptake in Denmark [9], as has the media attention with respect to the measles, mumps and rubella vaccine in the US [6]. For our quantitative analysis, we applied a similar idea. We used queries to extract news items from a news media database and search activity from Google. The news items were correlated with a time series of vaccination uptake to quantify a potential relationship. While the search activity was used as a way to quantify public attention on the HPV-vaccine and HPV-vaccine side effects.

## Methods

### Vaccination data

Childhood vaccinations are administered by general practitioners who report the vaccinations to the Danish Vaccination Register (DDV) [7]. We extracted data from 243,415 girls (retrieved October 2016) and used the data to calculate vaccination uptake by birth cohort as well as monthly vaccination activity (see below).

To quantify temporal effects of media on vaccination uptake we used the notion “vaccination activity”. We defined vaccination activity as the monthly number of girls who received the first dose of HPV-vaccine divided by the number of girls at the target age, i.e. girls turning 12 years that month. Dividing by the monthly birth cohort size controls for yearly variations in expected vaccination activity. In periods with high vaccination activity due to, e.g., catch-up activities, the vaccination activity can be higher than 100.

### Media coverage and public awareness

Retrospective analysis of media coverage is relatively simple due to the availability of comprehensive archives of published media, as will be discussed below. Data on public attention, on the other hand, is not readily available. In this study, we resorted to online search activity as a proxy for public attention. In a society with widespread access to the internet, search activity can be regarded as a measure of population behaviour, which is likely to be correlated with public attention.

The Google Health Trends service was used to download monthly search activity data. The service gives access to aggregated search data for a sample of 10–15% of the total number of searches. We restricted our sample to searches from Denmark. We extracted search activity for two searches: “hpv-vaccine” (HPV-vaccine) and “hpv bivirkninger” (HPV side effects/adverse events). The queries were chosen to distinguish between searches related to potential adverse events and searches reflecting a general interest in the vaccine. The Google Health Trends service allowed us to make a relative comparison between the search volume of different searches, though the absolute number of searches is unknown.

Media coverage was quantified as the number of published news items in a given month. News items matching a HPV-relevant query, see below (English translations in parentheses) was extracted from the media database, Infomedia [10]. The database covers nationwide newspapers, local newspapers, magazines, news agencies, web media, radio shows and TV shows [10].

*“HPV AND livmoderhalskræft (cervical cancer)“OR “HPV AND cervix cancer” OR “HPV AND cancer” OR “HPV AND kræft (cancer)” OR “HPV AND POTS” OR “HPV AND CRPS” OR “HPV AND kønsvorter (genital warts)” OR “HPV AND kondylomer (condyloma)” OR “HPV AND condyloma” OR “HPV AND sygdom (disease)” OR “HPV AND kønssygdom (sexually-transmitted disease)”.*

The query was designed to return as many HPV-related news items as possible. We excluded product names of HPV-vaccines (Gardasil and Cervarix) since we were uninterested in product specific effects. POTS (Postural Orthostatic Hypotensive Syndrome) and CRPS (Chronic Regional Pain Syndrome) are abbreviations commonly used in Danish media to describe suspected adverse events.

From Infomedia.dk we retrieved a total of 8524 unique news items, ranging from a minimum of 1 news item per month to a maximum of 507 news items in 1 month. An increase in news items matching the query was expected, as additional data sources, e.g. magazines or newspapers, are included in the database over time. To account for these changes, we normalized by the total number of news items containing the Danish word for “and”, on the assumption that all news items contain this word, this is referred to as the *media coverage*.

To confirm the initial hypothesis, i.e., that media coverage of the HPV-vaccine turned increasingly critical, a sample of news items were annotated into three categories: Focusing on benefits, neutral, or focusing on adverse events. For each year, the 20 news items that best matched the HPV query (according to Infomedias retrieval function) were selected. Three annotators independently performed the hypothesis-generating annotation of the sample of articles. Disagreement between annotators were solved using majority voting.

### Statistical analysis

To identify a potential change in the relationship between media coverage and vaccination uptake, we analysed changes in Pearson’s correlation. A temporal changing point is not a well-defined concept, and our choice of approach was based on the following reasoning. Pearson’s correlation quantifies to what extent the change in one signal coincides with changes in another. If media coverage and vaccination uptake were influenced by each other, we would expect a none-zero correlation. If the character of the relationship changed, we would expect the correlation to also change. We hypothesis that if the change happens quickly, we would be able to identify a changing point in time where the correlation changed for the period prior to the changing point compared with the period after. To assess this, we first calculated for each time point the correlation before and after, and subsequently we calculated the difference. Secondly, we calculated the derivative of this signal, i.e. the change between time points, and identified the time point with the largest change as the temporal changing point.

## Results

### HPV-vaccination uptake in Denmark

**Fig. 1:**
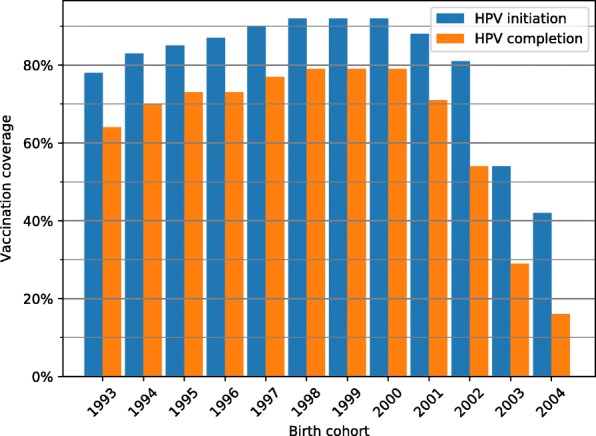
HPV-vaccination in birth cohorts 1993–2003, Denmark. HPV-vaccination initiation and completion for girls in the childhood vaccination programme, Denmark birth cohorts 1993–2003. Three-dose vaccination schedule from 2009 until August 2014. Two-dose schedule from August 2014 until 14 October 2016. Data extracted June 2017

Figure 1 shows that the vaccination uptake for the first dose of HPV-vaccine increased from 80 to 92% for the birth cohorts of 1993 to 2000. For girls born after 2000, the initiation of HPV-vaccination decreased for successive birth cohorts, reaching the lowest uptake of 42% for the birth cohort of 2004 (data per June 2017).

### Correlation between written media coverage and vaccination activity in Danish females

**Fig. 2:**
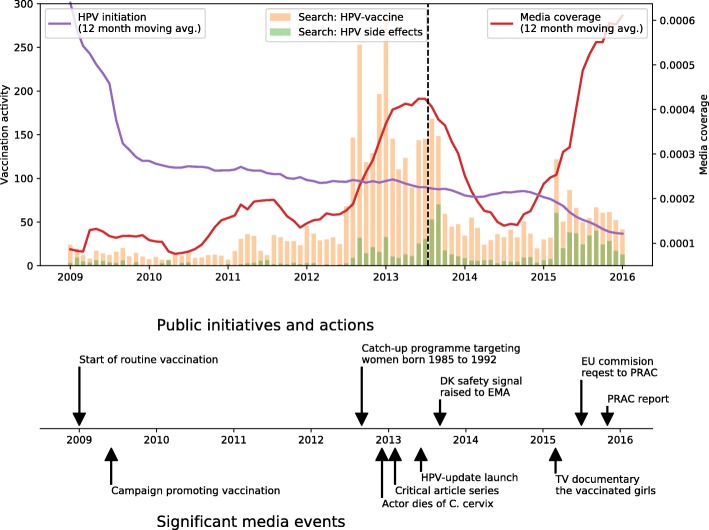
HPV-vaccination activity, media coverage, Google Health Trends search activity, public initiatives and significant media events, 2009–2016 in Denmark. Monthly HPV-vaccination initiation (HPV1) and media coverage in Denmark 2009–2015. Vaccination activity defined as proportion of vaccinated girls in relation to girls eligible for vaccination. Media coverage in Denmark 2009–2015 define number of HPV-related news items proportional to the total number of news items. Search activity indicates a relative probability for observation the defined search terms service allowing for a relative comparison between the search volume of different searches, though the absolute number of searches is unknown. The timeline in the bottom of the plot shows public initiatives and significant media events regarding the HPV-vaccine in Denmark. The vertical dotted line shows the changing point in the correlation between vaccine activity and media coverage

Figure 2 shows HPV-vaccination activity, HPV-related media coverage and search activity from January 2009 to January 2016. As illustrated in the figure there was a high vaccination activity in the first part of 2009 after the programme was launched. After the launch, activity remained quite stable until mid-2013 where the vaccination coverage dropped to below 90%. From the middle of 2012 to middle of 2013, there was a marked increase in both search activity and media coverage, which coincided with a drop in vaccination initiation. The public initiatives and significant media events regarding the HPV-vaccine are presented in chronological order in the bottom plot. The dotted vertical line marks the temporal changing point.

We identified July 2013 as the temporal changing point in correlation between media coverage and vaccination activity. Until June 2013 the correlation was non-significant (*r* = 0.18, *p*-value = 0.23), while it was significant and negative (*r* = − 0.52, p-value< 0.001) for the remaining period. The changing point occurred after the peak in media attention, i.e. July 2013, but before the peak in search activity for HPV side effects. While the correlation after the changing point is significant, a value of − 0.52 is not a strong correlation. Even if we assume a relationship between the two signals, a substantial proportion of the change in either signal must be attributed to other factors.

The time-line in Fig. 2 shows the public initiatives and selected significant media events regarding the HPV-vaccine in Denmark 2009–2013. A steep rise in media coverage and search activity was seen after the death of a young actor from cervical cancer in December 2012. In 2013, two series of critical articles in nationwide newspapers questioned the reliability of professional recommendations and later suspected adverse events. Several patient organisations were formed, such as “HPV-Update” under the Danish Association of the Physical Disabled, comprising parents believing that their daughters suffered from adverse events after HPV-vaccination [11] . A TV documentary from March 2015, “De vaccinerede piger“ (The Vaccinated Girls) displayed a group of girls reporting a diverse array of symptoms perceived as adverse events following HPV-vaccination [12]. This documentary was widely discussed in the media and on social media platforms, coinciding with increased search activity. In the same period, the chairperson of the Health Committee in the Danish Parliament voiced great concern for the safety of the HPV-vaccine [13]. The issues of vaccination safety kept resurfacing in the media, an important driver was the freely available newspaper MetroXpress that had a daily feature page along with a webpage on the “HPV case” (Danish: HPV-sagen) [14]. The Danish safety signal was raised to the European Medicines Agency (EMA) Pharmacovigilance Risk Assessment Committee (PRAC) in September 2013. In July 2015, the European Commission requested PRAC to assess whether there was evidence of a causal association between HPV-vaccination and POTS and/or complex regional pain syndrome (CRPS). The suspicion of such an association had also been raised from Japan [15]. PRAC concluded that no current evidence supported that HPV-vaccination causes CRPS or POTS [16]. In December 2015, the WHO Global Advisory Committee on Vaccine Safety reported that it had not found any safety issues that would alter its recommendation [17]. The same year, the Danish National Board of Health reaffirmed the favourable risk/benefit balance of HPV-vaccination in a public statement [3].

**Fig. 3:**
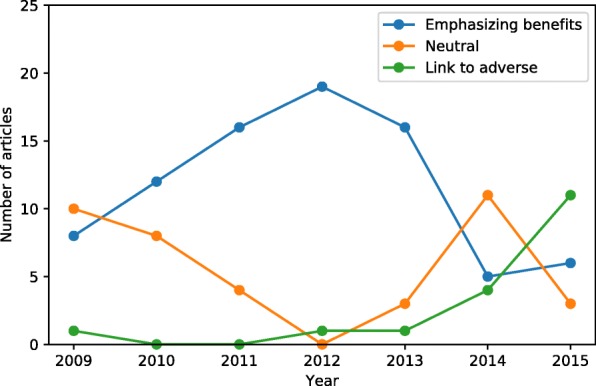
Sentiment annotation of 140 articles. The yearly distribution of annotated articles. The overall raw agreement was 85.4% between the three independent annotators

Figure 3 shows the distribution of the sentiment-annotated articles. The number of articles focusing on benefits peaked in 2012 with 19/20 articles, after which it slowly dropped to 6/20 in 2015 with 11/20 articles focusing on a potential link to adverse reactions.

## Discussion

### Introduction of the HPV-vaccine in Denmark

After a successful launch of the HPV-vaccination programme, the vaccination uptake remained high for 6 years, after which we saw a decline. In this paper we describe the events leading up to the decline and focus on the potential correlation with media coverage and on-line search activity as a proxy of public attention. Our analysis of the correlation shows a change in the relation between the media coverage and vaccination uptake in July 2013.

The findings could indicate that as media attention increased, the public confidence in the safety of the vaccine decreased, and accordingly, vaccination uptake was reduced. The situation was self-perpetuating with a continuous stream of news-stories describing the decline in HPV-vaccination uptake and suggesting fear of adverse events as leading explanation. Media coverage related to the HPV-vaccination increased from 2012 and peaked in June 2013. Initially, the media attention was focusing on the benefits of vaccination, i.e. cancer prevention, which was also reflected in the annotated articles (see Fig. 3) and coincided with an increase in search activity on HPV-vaccine (see Fig. 2). The decline in vaccination activity stabilised in 2014 but was accelerated after the airing of the documentary “The vaccinated girls” in March 2015. In the same period, the search activity on adverse events increased markedly, indicating a profound impact on the public risk perception of the HPV-vaccine. There was also a noticeable increase in the number of reported adverse events related to HPV-vaccination in Denmark from 2012 with 95 reports compared to 2015 with 821 reports [4].

The effect of media coverage on vaccination uptake has been studied in other contexts. Influenza vaccination rates has been shown to increase after significant media attention to a severe influenza season in the US [18]. Further, articles suggesting a link between the MMR vaccine and autism has been linked to a drop in MMR vaccination in the US [6] and the UK [19]. A recent Danish study on the correlation between media coverage and the MMR vaccine showed that controversies in the media coincided with a correlation between vaccination uptake and media coverage, indicating a potential relationship [8]. Our hypothesis about the connection between increased adverse event reporting and media coverage is corroborated by other studies. Eberth et al. reported that media coverage and in particular internet search activity may stimulate increased adverse event reporting [20]. Faase et al. found results suggesting adverse events reporting was not related to the HPV-vaccination but to the news coverage and internet search stimulating concerns [21]. Dunn et al. used Twitter to derive media exposures and showed that HPV vaccination coverage was lower in areas with higher proportion of exposure for safety concerns, misinformation and conspiracies [22].

The effect of case stories  reported in the news has not only been seen in Denmark. In Ireland concerns are raised as fewer girls receive HPV-vaccine [23, 24]. The Japanese government suspended their active recommendation of the HPV-vaccine in 2013 in response to unfounded fears about the safety profile. The decision is still unreversed despite a supportive declaration from the country’s Vaccine Adverse Reactions Review Committee [25]. Apart from the withdrawal of the HPV-vaccine in Japan, it is our impression that no other country had yet to experience an HPV crisis as strong as in Denmark. However, the proportion of girls who completed the vaccination schedule in Ireland dropped to an estimated uptake of 50% for the first dose in 2016–17 [26]. The fact that other countries had yet to experience an HPV crisis as strong as in Denmark makes   an influence from non-Danish media sources on the vaccination choices less likely. Karafillakis et al. have investigated vaccine-safety scepticism and found, with the notable exceptions of France and Italy, that Western and Northern European countries express less concern about vaccine safety than Eastern and Southern European countries [27].

Our data provide indirect insight into the decision process to vaccinate ones child. This decision is influenced by multiple factors, one of those being publications in the media [28]. Media is part of shaping parents impressions of the safety of a vaccine, which is the largest concern parents have today regarding vaccines [29]. A greater belief in the protection offered by childhood vaccines has been found to correlate with acceptance of HPV-vaccines [30]. Parent socio-economical [31] and social-environmental factors including cultural beliefs as well as social group norms may also play a role [28]. This influence of social group norms could explain the rapid decline in vaccination seen in our study. Both individual factors, as well as social group norms, can be influenced by massive media coverage and impact parental vaccination choices through different pathways. In other countries, such as the UK and US, parental concerns over HPV-vaccination promoting promiscuity [32]. This has not been a topic in the Danish debate [33].

In addition to the previously mentioned factors, physicians play a pivotal role in the parental vaccination decision pathway. Daley et al. has reported that physicians attitudes and intentions of recommending HPV-vaccination promote successful immunization delivery [34]. Gilkey et al. found that providers presented HPV as an “optional” vaccine that can be delayed [35]. HPV-vaccination being the latest addition to the Danish vaccination programme places it in a vulnerable position since physicians perceive the most recently introduced vaccines in the programme to be less important compared with the former [36].

Monitoring media activity and describing the effect of media coverage on vaccination may help to develop new approaches to reach and maintain the optimal vaccination uptake. This is also very important to keep in mind when planning the introduction of potential new vaccines into a childhood vaccination schedule. Listening to the public should be a fundamental element of any introduction of a new vaccine. Larsson et al. have constructed a communication model that envisions communication as integrating safety assessment and trust-building strategies [37]. Bahri et al. showed how real-time global media monitoring could be used for enhancing communication proactivity and preparedness to support vaccine use [38]. In Denmark, the National Health Authority together with the Danish Cancer Society and other stakeholders launched an informational campaign, 2017, using as the key platform a Facebook page where professionals engage with the public in a timely manner [39]. The Facebook page also directs traffic to an information homepage www.stophpv.dk [40] with additional material. A similar multi-stakeholder approach has been successful in raising the uptake of HPV-vaccination in Ireland [26].

### Strength and limitations

Using the DDV, we are able to closely monitor the development in HPV-vaccination uptake on population level using person identifiable data on a real time scale [7]. The validity of the DDV has previously been studied indicating a 3 percentage points underestimation [41]. The reporting to the DDV has since been made mandatory and automatized which should improve the completeness of the register [42]. Our study was ecological in its design, limiting the possibility of causal inference.

While the media archive used for the media coverage analysis covers all the major Danish news sources, it is still dynamically changing which might introduce a bias. The most vocal media in the HPV debate have been present in the archive throughout the study period and bias through changes in the database content should therefore be limited. It is evident from the case of the documentary “The vaccinated girls” that a lot of spin-off stories were published in the written media. A large percentage of Danish parents may have sought their information online or in social networks since 92% of Danes have access to the internet at home [43]. Not having data on social media is a limitation in this study and is an area for further research. However, Mollema et al. identified a strong correlation between the number of messages on social media and online news articles, indicating that public opinion is reflected on these platforms and that the written media determine themes discussed on social media [44].

We analysed the content of a sample of the media stories and found the results to confirm our prior perception of increasingly negative media sentiment (see Fig.3). Previous studies have shown that just the mention of a controversy in the media may impact public awareness regardless of specific content [45]. This paper is intended as a description of the events leading up to the decline in the HPV-vaccination uptake.

## Conclusion

The HPV-vaccination uptake was very high in Denmark for the first years after the introduction in the childhood vaccination schedule. Following public concern and media attention, the uptake dropped to a dissatisfactory level of 54% for the girls born in 2003. The drop in vaccination uptake of HPV-vaccine correlated with written media attention from July 2013.

This study offer important information for the public health community as it continues to work for higher acceptance of present and emerging vaccines. Our findings suggest that media could have a potential impact on vaccination uptake. If providers and parents become more cautious during periods of controversy, it will be very important that the health authorities are aware of this. For many parents, vaccination decision-making is complex and providing recommendations from authorities is not enough to safeguard a vaccination programme. Close monitoring of media and public sentiment might allow for early detection of emerging problems enabling timely action, and this information can be used in shaping communication activities. Media coverage has to be taking into account when planning public health interventions including introduction of a new vaccine. A strategy for handling a sudden change in public opinion reflected in media could prove vital for reaching and maintaining the optimal vaccination uptake. WHO has developed an e-learning module for crisis communication including a case study on how a potential HPV-vaccine crisis was averted in the UK [46]. Allegations regarding vaccine-related adverse events needs to be dealt with rapidly and effectively to not undermine confidence in the vaccine. Managing inaccurate perceptions of vaccination risks is as important as handling scientifically confirmed risks [37]. From a public trust standpoint, it is advisable to proactively take control of the story by communicating rapidly, accurately and provide transparency [46].

## Abbreviations

CRPS: Chronic Regional Pain Syndrome
DDV: the Danish Vaccination Register
EMA: European Medicines Agency
HPV: Human Papilloma Virus
MMR: Measles, Mumps and Rubella
POTS: Postural Orthostatic Hypotensive Syndrome
PRAC: Pharmacovigilance Risk Assessment Committee
WHO: World Health Organization
